# RES-MND: Motor neuron disease detection using Res4Net–convolutional block attention module

**DOI:** 10.55730/1300-0144.6177

**Published:** 2025-10-03

**Authors:** Shuriya BALUSAMY, Rakesh SIVALINGAM, Shobana ROOBEN, Sony HELEN

**Affiliations:** 1 Department of Computer Science and Engineering, United Institute of Technology, Coimbatore, India; 2 Department of Computer Science and Engineering, Aurora Higher Education & Research Academy - Deemed to be University Bhongir, Hyderabad, Telangana, India; 3 Department of Computer Science and Engineering, S. A. Engineering College, Chennai, India; 4 Department of Computer Science and Engineering, Arunachala college of engineering for women, Manavilai, Vellichanthai, India

**Keywords:** Motor neuron diseases, adaptive dynamic histogram equalization, total variation bilateral filter, Res4Net-CBAM, deep belief network

## Abstract

**Background/aim:**

Motor neuron diseases (MNDs) are progressive neurological disorders that cause muscle weakness and wasting as a result of ongoing neurodegeneration. MNDs require comprehensive diagnostic approaches that integrate clinical symptoms, laboratory findings, and multimodal imaging data.

**Materials and methods:**

In this study, a novel residual network for motor neuron disease detection (RES-MND) framework is proposed for detecting MNDs using multimodal imaging data. Initially, the input multimodal images, including MRI, CT, PET, and DTI, are preprocessed using adaptive dynamic histogram equalization and the total variation bilateral filter. The preprocessed multimodal images are processed through Res4Net-CBAM for feature extraction to enhance image recognition performance. A dove swarm optimization algorithm is employed to select the most relevant features from the multimodal images. Finally, the deep belief network (DBN) classifies five categories, including one control group (normal) and four MND types: ALS, PLS, PBP, and PMA.

**Results:**

The performance of the proposed RES-MND method is evaluated using standard metrics such as accuracy, precision, recall, and F1-score. According to the results, the proposed RES-MND method achieved the highest accuracy rate of 99.65%, outperforming existing methods.

**Conclusion:**

The proposed DBN achieved 0.68%, 0.41%, and 0.9% higher accuracy than SNN, DNN, and CNN, respectively. The proposed RES-MND method achieved 1.08%, 2.18%, and 1.7% higher overall accuracy compared to existing methods such as miRNA, vGRF, and SVM-RFE, respectively.

## 1. Introduction

The human brain is the most complex and integral organ of the human body. One of the brain’s most vital roles is regulating the central nervous system [1,2]. The human brain primary functions include thinking, moving, coordinating, visualizing creatively, learning, remembering, and responding emotionally [3]. The brain controls the entire human body, and any abnormality in its function can disrupt or even paralyze normal physiological processes [4]. MNDare progressive neurodegenerative disorders, including amyotrophic lateral sclerosis (ALS), that damage both upper and lower motor neurons (UMN and LMN) [5]. A “motor band sign” (MBS), characterized by a hypointense signal along the precentral gyri, is frequently observed on magnetic resonance imaging (MRI) in ALS and is considered a characteristic radiologic finding [6,7]. The hippocampal region, located deep within the temporal lobe and associated with memory, learning, and emotion, is an essential component of the brain [8]. MRI remains limited in its diagnostic utility for individuals suspected of having ALS, primarily serving to rule out alternative causes of motor neuron dysfunction [9]. Researchers analyzed data from the UK Biobank, which has collected DNA samples from 500,000 individuals. This condition has been found to be more prevalent among individuals with genetic profiles associated with high physical activity [10,11].

Diagnosing MNDs is challenging because their symptoms often overlap with those of other neuromuscular disorders [12,13]. Early diagnosis is essential for implementing multidisciplinary treatment strategies aimed at managing symptoms and improving patients’ quality of life. MNDs have no known cure; therefore, therapeutic approaches focus on alleviating symptoms and slowing disease progression [14–16]. The progressive weakening of spinal muscles occurs gradually and is not immediately fatal; however, motor neuron diseases generally lack effective treatment [17,18]. To address these challenges, a novel residual network for motor neuron disease detection (RES-MND) model is proposed for detecting MNDs using multimodal imaging data.

- Initially, the input multimodal images, including MRI, CT, PET, and DTI, are preprocessed using adaptive dynamic histogram equalization (ADHE) and the total variation bilateral filter (TVBF).
- The preprocessed multimodal images are processed through the Res4Net-CBAM architecture for feature extraction to enhance image recognition performance.
- A dove swarm optimization algorithm is employed to select the most relevant features from the multimodal images.
- Finally, the deep belief network (DBN) classifies five categories, including one control group (normal) and four MND types: ALS, primary lateral sclerosis (PLS), progressive bulbar palsy (PBP), and progressive muscular atrophy (PMA).

There may be a shortage of MND records, particularly longitudinal data that illustrate disease progession over time. Numerous studies have discussed the latest developments in deep learning (DL) techniques.

In 2020, Banack et al. [19] identified neural-enriched extracellular vesicles showing clear indications of neurodegeneration in their microRNA (miRNA) profiles. A total of eight miRNA sequences showing differential expression between healthy individuals and ALS/MND patients were identified through repeated testing across multiple patient and control cohorts. This replication provides compelling evidence that these miRNA sequences, either individually or in combination, warrant further investigation as potential ALS/MND biomarkers in larger sample sizes. It is also essential to compare these findings with those from other neurodegenerative diseases. In 2021, Setiawan and Lin [20] proposed an innovative deep learning–based identification technique for neurodegenerative disease (NDD) classification using vertical ground reaction force (vGRF) signals. The primary goal of their study was to assist clinicians in effectively planning treatments, enabling early NDD detection, and monitoring disease progression. The proposed detection technique effectively distinguishes between healthy controls (HC) and patients with neurodegenerative diseases using the time–frequency spectrogram of the vGRF signal. In 2021, Greco et al. [21] reported that numerous motor neuron disorders (MNDs) share overlapping symptoms, making early differential diagnosis challenging. Patients were automatically classified into either the ALS or LMND categories using support vector machine with recursive feature elimination (SVM-RFE), which also determined whether the disease progressed rapidly or gradually. The classification method using the RFE algorithm also identified a subset of blood analytes that offered the greatest insight, including novel potential MND biomarkers. In 2022, Sekar et al. [22] presented a neural machine learning model designed to detect motor neuron disease and predict its potential impact on health. The machine learning system utilizes various symptoms to predict the effects of motor neuron disease. A speech therapist can recommend assistive communication devices and mobility aids while addressing orthopedic stability. The proposed approach achieved prediction accuracies of 93.28% for bulbar palsy, 91.44% for tendon degradation, 93.22% for polytopic paralysis, and 92.12% for amyotrophic lateral sclerosis based on the specified symptoms. In 2022, Bede et al. [23] proposed a multiclass machine learning approach to assign diagnostic labels to 300 patients across the UMN-LMN spectrum based on their radiological profiles. Large training datasets are required to interpret radiological data from individual patients, and providing diagnostic probability estimates for those with early symptoms could be clinically beneficial. Computational interpretation of multimodal radiology datasets represents a promising approach for diagnosing and prognosing patients, as well as supporting clinical trials. In 2024, Vernikouskaya et al. [24] proposed a convolutional neural network (CNN) model to measure tongue volume using MRI data. Tongue regions in head MRI scans were segmented using a single triplanar CNN based on the U-Net architecture, trained on axial, coronal, and sagittal planes. The predictions were combined using multiple voting techniques following slice-wise processing of the 3D volumes across all three orientations. In 2024, Zhang [25] developed a deep learning–driven genomic research framework to link rare noncoding genetic variants to ALS survival. This approach employs deep learning to identify functional noncoding variants and discovers survival-modifying variations by integrating epigenomic data that link cis-regulatory elements (CREs) to target genes, combined with gene-level burden testing. Single-cell methods are frequently used to map molecular heterogeneity and cellular diversity across various biological contexts.

Several deep learning techniques have been employed in the literature to detect motor neuron diseases. The RES-MND method is proposed to improve accuracy while minimizing computational complexity.

The remainder of this study is structured as follows. Section 2 presents the relevant studies in detail; section 3 provides a comprehensive explanation of the proposed RES-MND model along with the experimental results; and section 4 discusses the findings and their implications.

## 2. Materials and methods

**Figure 1:**
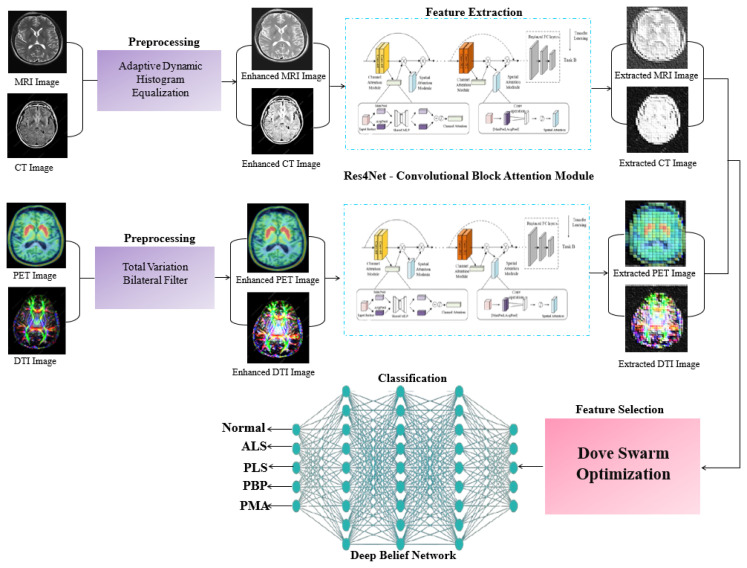
Overview of the proposed RES-MND method.

In this section, a novel RES-MND model is proposed for detecting MNDs using multimodal imaging data. Figure 1 illustrates the architecture of the proposed RES-MND model.

### 2.1. Image preprocessing

In this section, the multimodal input images are preprocessed to enhance their visual quality. The adaptive dynamic histogram equalization (ADHE) technique is employed to preprocess the input CT and MRI images. The total variation bilateral filter (TVBF) is then applied to preprocess the PET and DTI images, further enhancing the overall quality of the multimodal images.

#### 2.1.1. Adaptive dynamic histogram equalization

This method computes the peak values of the image histogram and forms clusters based on these peaks. This process is iteratively performed until the image no longer contains clustered regions. Histogram-based equalization is more effective than other enhancement techniques and needs to be applied only once to each pixel. The first step in ADHE involves smoothing each histogram to reduce noise and fluctuations. Adjacent pixels are then compared to identify local maxima within the histogram. The smoothing process eliminates jagged peaks caused by high-frequency noise components in the image. By adjusting pixel intensities, this method enhances specific features of the image. The smoothing process, which increases or decreases pixel intensity, employs a Gaussian function. In the Gaussian function, each pixel is transformed according to a normal distribution to reduce image blur. The Gaussian function is defined as follows:


(1) $$G(h,v)=\frac{1}{2\pi \sigma^{2}}e^{\frac{-h^{2}+v^{2}}{2\sigma^{2}}}$$

where σ denotes the standard deviation, h represents the horizontal axis distance, and v corresponds to the vertical axis distance. Thus, the flattened image becomes suitable for contrast enhancement (CE). This function eliminates extreme and redundant noise peaks, including both maximum and minimum values. The smoothing of maximum points in the region of contrast (ROC) separates the darkest and brightest areas of the image. A local maximum represents the highest point of the histogram relative to its neighboring values. This makes it easier to distinguish between the brightest and darkest intensity levels. The smoothed image histogram traces the local maximum and minimum points. The image’s highest and lowest intensity values are used to determine local maxima and minima. The lowest intensity value is represented by 0, and the highest by 255. The image is partitioned according to its highest and lowest intensity levels. In this case, the image histogram is used to compute the median intensity value. The median is calculated using the following equation:


(2) $$I_{median}=L_{m}+[\frac{\frac{N}{2}F_{m-1}}{f_{m}}]\;C$$

where *L_m_* is the lower median value, *N* is the total number of observations, *f_m_*_-1_ is the cumulative frequency, *fm* denotes the frequency of the median class, and C represents the class interval. The median value is subsequently used to segment the image. The interval refers to the separation between two consecutive local maxima. For faster analysis, partitioning is required to group correlated pixel values simultaneously.

#### 2.1.2. Total variation bilateral filter

Total variation–based denoising effectively reduces noise while better preserving image edges. False edges may appear when noisy pixels in flat regions are mistakenly interpreted as edge pixels. An adaptive regularization term is desirable to enhance denoising performance while preserving edges and structural details. The bilateral filter is well known for its capability to smooth images while preserving edge information. To overcome the limitations of total variation, the TV regularization term is replaced with a bilateral filtering term. The bilateral filter is chosen as the TV regularization term for the following reasons: (1) TV alone cannot simultaneously smooth and preserve feature information during denoising; therefore, a term capable of preserving edges must be introduced, and the bilateral term satisfies this condition. (2) In TV, the regularization term provides a unique solution; similarly, the bilateral term yields a unique solution as well. This section explains the proposed algorithm, starting with the modeling of speckle noise. The multiplicative speckle noise can be modeled as follows:


(3) f(x,y)=T(x,y)η(x,y)

where *T*(*x,y*) is the noise-free image, *f*(*x,y*) is the acquired image, and *n*(*x,y*) represents the multiplicative noise. Here, *x* and *y* denote the spatial coordinates. This process involves converting the logarithm of the input image from multiplicative to approximately additive noise.


(4) log[f(x,y)]=log [T(x,y)η(x,y)]

(5) =log [T(x,y)]+log [η(x,y)]

The above equation can be rewritten as:


(6) $$f_{xy}=T_{xy}+\eta_{xy}$$

where *f_xy_* is the noisy image and *T_xy_* represents the true signal, which the extreme total variation bilateral (ETVB) model uses and retains as prior information. The ETVB integrates the previous term and replaces the regularization component of the total variation approach with the bilateral filtering term to effectively denoise the image. The following equation describes how the total variational approach eliminates noise from an image:


(7) $$u_{den}=argmin_{u}\int_{\Omega }{\left(u-f\right)}^{2}dx+\lambda$$

where *u*_den_, *f* and *λ* denote the denoised image, the input image, and the smoothing factor, respectively.

Equation (7) is solved using the gradient descent and Euler–Lagrange optimization methods, resulting in the formulation shown in Equation (8).


(8) $$\frac{\left(f-u\right)}{\sigma_{fu}^{2}}+\lambda *div\;\left[\frac{\nabla u}{|\nabla u|}\right]=0$$

Here σ*_fu_* defines the Gaussian range of *f* within which *u* can vary, while *div* [∇*u*/|∇*u*|]] represents the regularization term. The bilateral filtering component, denoted as ũ(*k*) is substituted into this equation to yield the following expression:


(9) $$\tilde{u}(k)=\frac{\sum_{p\in N(k)}G_{c}(\left||p-k|\right|)\times G_{s}(\left||u(p)-u(k)|\right|)\times u(p)}{\sum_{p\in N(k)}G_{c}(\left||p-k|\right|)\times G_{s}(\left||u(p)-u(k)|\right|)}$$

Here, *G_c_*(||*p*-*k*||) is the closeness (spatial) smoothing function $e^{-x^{2}/2\sigma_{c}^{2}}$, and *G_s_*(||*u*(*p*)- *u*(*k*)||) is the feature-preserving weighing function $e^{-x^{2}/2\sigma_{c}^{2}}$, which penalizes large intensity variations. For each pixel *k* in the noisy image, *N*(*k*) represents the neighborhood around *k*. Substituting Equation (9) into Equation (10) yields the following modified denoising formulation:


(10) $$\frac{\left(f-u\right)}{\sigma_{fu}^{2}}+\lambda +\tilde{u}(k)=0$$

The conventional TV approach incorporates the concept of prior knowledge, which refers to general information about the object being analyzed.

### 2.2. Res4Net–convolutional block attention module for feature extraction

**Figure 2:**
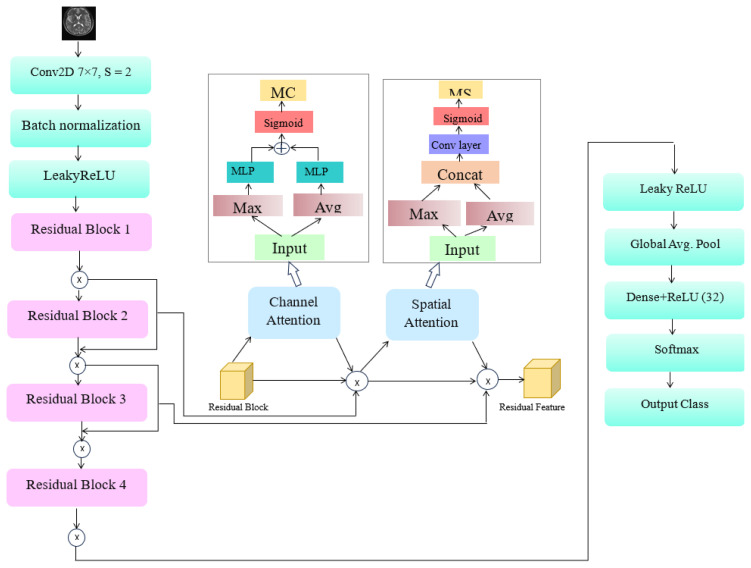
Architecture of the proposed Res4Net-CBAM model.

The Res4Net-CBAM model is designed for feature extraction to enhance image recognition performance. LeakyReLU activation and batch normalization (BN) layers are incorporated to construct a transition layer that regulates input flow and accelerates training convergence. The residual (convolutional) blocks consist of stacked 2D convolutional and batch normalization layers. The transition layer is followed by either an identity or convolutional skip connection. The Res4Net classification network is constructed using four enhanced residual block modules. A global average pooling operation flattens and compresses the high-level feature maps following the residual blocks. A fully connected layer, followed by an output layer and dropout, is used to obtain the final class prediction. The attention mechanism, a deep learning technique, focuses on salient and automatically learned features. The attention model allocates computational resources primarily to the most informative features. The channel attention module (CAM) and the spatial attention module (SAM) are two submodules of convolutional block attention module (CBAM). The CBAM module is compatible with most existing architectures and reduces computational cost and parameter complexity. The channel and spatial attention modules are sequentially integrated into the input and output stages of the CBAM structure. The CBAM combines spatial information using two pooling strategies: global maximum pooling and global average pooling. Combining these two pooling operations ensures comprehensive high-level feature extraction and eliminates redundant data, enabling precise learning of interchannel dependencies. The channel attention module highlights the most important image features while suppressing irrelevant details. The first step processes the input features concurrently using both max pooling and average pooling operations. These two types of information are then passed through a single hidden layer of a multilayer perceptron. Finally, component-based aggregation is applied to combine the resulting feature representations. The architecture of the Res4Net-CBAM model is illustrated in Figure 2.

The channel attention module (CAM) is mathematically expressed as follows:


(11) $$\begin{array}{l} M_{c}(F)=\sigma (MLP\;(AvgPool(F))+MLP(MaxPool(F))\\ =\sigma \left(W_{1}\left(W_{0}(F_{avg}^{c})\right)+W_{1}(W_{0}(F_{max}^{c}))\right) \end{array}$$

where *M_c_* denotes the channel attention map, *F* represents the input features, σ is the sigmoid activation function, and *W_1_* and *W_0_* are learnable weight matrices. During spatial attention module (SAM) processing, the regions selected by the channel attention module are considered the most relevant input features. The output features are processed by a convolutional layer after being concurrently handled by max pooling and average pooling operations. The spatial attention module (SAM) can be expressed as follows:


(12) $$M_{s}(F)=\sigma (f[AvgPool(F);Max\;Pool(F)])$$

where *M_s_* denotes the spatial attention map, and *f* represents the convolution operation. The input to the network consists of 128 × 128 images with three depth-based color channels (R, G, and B). The input image is processed by the first layer of the Res4Net-CBAM, which is a 7 × 7 convolutional layer with a stride of 2 × 2. The subsequent layers consist of LeakyReLU activation and batch normalization (BN), which primarily function as a transition layer to regulate inputs and accelerate training. To more precisely extract image features, the output of each residual block in the intermediate layer is passed through the CBAM module.

The output of the CBAM module is multiplied by the output of the initial residual block and then added to the subsequent residual block. A single fully connected layer with 32 neurons follows a dropout layer with a 30% dropout rate. The final layer employs a softmax activation function for multiclass classification. The softmax function is defined as follows:


(13) $$P(y=i|X;\theta )=\frac{\text{exp}\left(\theta_{i}^{T}X\right)}{\sum_{k=1}^{k}\text{exp}\left(\theta_{i}^{T}X\right)}$$

where *x* is the input vector, *⊝i* is the parameter vector corresponding to class *i*, and *yi* denotes the class label. The probability of each output *yi* given input x is represented by P(*y*=*i|X; ⊝*).

### 2.3. Dove swarm optimization

The proposed dove swarm optimization algorithm for feature selection is inspired by the foraging behavior of doves and is designed to identify the most relevant features from multimodal images. In nature, doves feed in open spaces where crumbs are scattered, and each dove actively searches for food particles. Some unsatisfied doves may fly ahead to nearby locations in search of additional food sources. Over time, it becomes evident that well-fed doves consume most of the available food resources. This natural foraging behavior has inspired the development of a novel optimization algorithm based on dove swarm dynamics.

**Figure 3:**
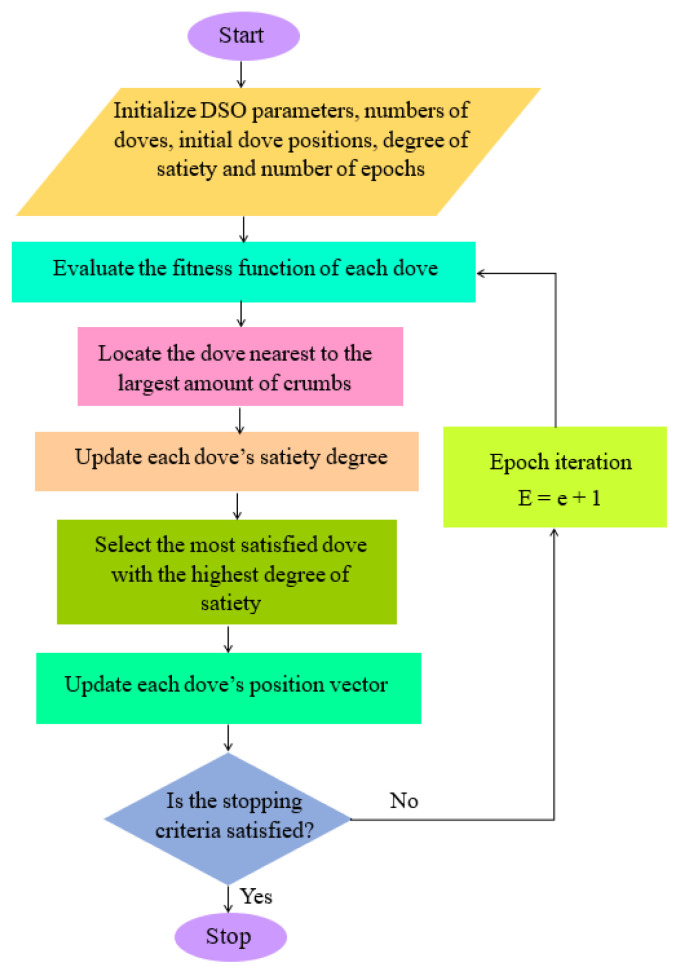
Flow diagram of the dove swarm optimization algorithm.

In this approach, the optimization objective function is defined as *f* (*W*). Each data pattern *W* represents a location in the search space containing a certain number of crumbs, analogous to the quantity of features in that region. The fitness value *f* (*W*) corresponds to the quantity of crumbs (i.e. the objective value) at location *W*. The optimal location corresponds to the area with the highest amount of crumbs, representing the best fitness value. Before initialization, a predefined number of doves is generated and distributed across the solution space. Let the total number of doves be denoted as N. These doves are initially distributed uniformly across a rectangular search area, although random dispersion may also be applied. The flow diagram of the dove swarm optimization algorithm is illustrated in Figure 3.

The cells located at the four corners of the network are initialized first, with their weight vectors set to unity.


(14) $$w_{1,1}={\left(l_{1},l_{2},\ldots \;\ldots \;\ldots ,l_{M}\right)}^{T}$$

(15) $$w_{A,B}={\left(u_{1},u_{2},\ldots \;\ldots ,u_{M}\right)}^{T}$$

(16) $$w_{1,B}={\left(l_{1},l_{2},\ldots \;.,l_{\left\lfloor \frac{M}{2}\right\rfloor },u_{\left\lfloor \frac{M}{2}\right\rfloor +1,\ldots \ldots .,}u_{M}\right)}^{T}$$

(17) $$w_{A,1}={\left(u_{1},u_{2},\ldots \;.,u_{\left\lfloor \frac{M}{2}\right\rfloor },l_{\left\lfloor \frac{M}{2}\right\rfloor +1,\ldots \ldots .,}l_{M}\right)}^{T}$$

The learning rate is initially set to 0.1 and decreases over time according to the following equation:


(18) $$\eta (n)=\eta_{0}\times \left(1-\frac{t}{T}\right)=0.1\left(1-\frac{t}{100}\right)$$

where *t* denotes the iteration number, and η_0_ represents the initial learning rate. The computed fitness function of the doves, $\text{f}(w_{j}^{e})$, j=1, ....,N represents the total number of crumbs at the position of the *d^th^* dove during iteration *e*. The dove $d_{j}^{e}$ that achieves the highest number of crumbs at epoch *e* is determined by the following maximum criterion:


(19) $$d_{j}^{e}=\text{argmax}\left\{f(w_{j}^{e})\right\},\;for\;j=1,\ldots \;\ldots ,N$$

The satisfaction level of each dove is updated using the following equation:


(20) $$S_{j}^{e}=\lambda S_{j}^{e-1}+e^{\left(f(w_{j})-f(wd_{f})\right)},for\;j=1,\ldots \;\ldots ,N$$

The dove with the highest satisfaction level, $d_{s}^{e}$ is determined using the following maximal criterion:


(21) $$d_{s}^{e}=\text{arg}\underset{1\leq j\leq N}{\text{max}}\left\{S_{j}^{e}\right\},\;for\;j=1,\ldots \;\ldots ,N$$

The dove *ds* selected according to Equation (22) is the best-performing dove in the flock in terms of foraging ability and serves as the reference for replication. The following maximum criterion is applied to update the position vector of each dove:


(22) $$w_{j}^{e+1}=w_{j}^{e}+\eta \beta_{j}^{e}(w_{d_{s}}^{e}-w_{j}^{e})$$

where


(23) $$\beta_{j}^{e}=\left(\frac{S_{b_{s}}^{e}-S_{j}^{e}}{S_{b_{s}}^{e}}\right)\;\left(1-\frac{||w_{j}^{e}-w_{d_{s}}^{e}||}{maxDistance}\right)$$

(24) $$maxDistance:\underset{1\leq j\leq N}{\text{max}}||w_{j}-w_{i}||$$

The parameter η represents the learning rate used to update the dove’s position vector. Equations (22)–(24) provide a detailed explanation of the position update mechanism in the subsequent iteration. The termination condition of the dove swarm optimization algorithm is defined as follows:


(25) $$|f_{d_{s}}^{e}-T(e)\leq eore\leq theset\;\text{max}\;epoch$$

The computational complexity of the dove swarm optimization algorithm is *O* (*NN_d_e*), where *N_d_* is the number of data points in the dataset, *N* is the number of doves, and *e* denotes the number of epochs. This can be expressed as a linear order of complexity with respect to *N,N_d_*, and *e*.

### 2.4. Deep belief network for classification

A deep belief network (DBN) is a type of deep learning model composed of multiple layers of restricted Boltzmann machines (RBMs). In a DBN, deep hierarchical structures are formed by stacking RBMs on top of one another. The DBN framework associates the number of data features with the input layer size and the number of output nodes with the corresponding target categories. The input data distribution is modeled using hidden variables within a statistical generative framework designed through the DBN architecture. The DBN employs a two-stage learning process. The first stage involves greedy layer-wise pretraining of each RBM. At this stage, each RBM learns to identify fundamental patterns in the data while temporarily ignoring the overall network complexity. The subsequent stage fine-tunes the entire network to minimize the reconstruction error between the original input data and the data reconstructed by the DBN. Although computationally intensive, this process significantly enhances the model’s representational capability and performance.

It can be used to compute probability distributions across hidden and visible units, where *u* and *l* denote the hidden and visible layers, respectively, *Y* (or *Z*) represents the partition function, and *A*(*u, l*) corresponds to the energy function.


(26) $$D(u,l)=\frac{1}{Y}\text{exp}\left(-A(u,l)\right)$$

The principal feature of the DBN is its weight matrix *U*, which is determined by the underlying RBMs and is learned through the conditional probability p (w*|f,U*). The posterior mean for hidden units is represented by *q (f|w)*. Equation (27) expresses the rate at which the visible layer gradient is computed.


(27) $$P(w)=\Sigma f(p\left(\frac{f}{U}\right)p\left(\frac{w}{f},U\right))$$

The vector *sum(z_d_)* which connects all hidden layers of the DBN, is formulated as follows:


(28) $$sum(z_{d})=\Sigma_{c_{j}=1}^{m}num(fdc_{j})$$

Equation (29) represents the output of the hidden layer as a function of the image weights and biases.


(29) $$A^{d}=[\Sigma_{m=1}^{l}Z_{md}^{G^{t}}*p_{m}]A_{d}\forall p_{m}=e_{m}^{2}$$

In this context, *P_m_* denotes the output of the RBM layer used for the multilayer classification process, while *A^d^* represents the hidden layer bias corresponding to the input data.

## 3. Results

In this section, the experimental evaluation of the proposed model is implemented using MATLAB-2020b to analyze its overall effectiveness. The proposed model was evaluated using both publicly avaiable data from the Kaggle dataset and real-time patient data collected from the SAM Neuro Center, Kanyakumari.

**Figure 4:**
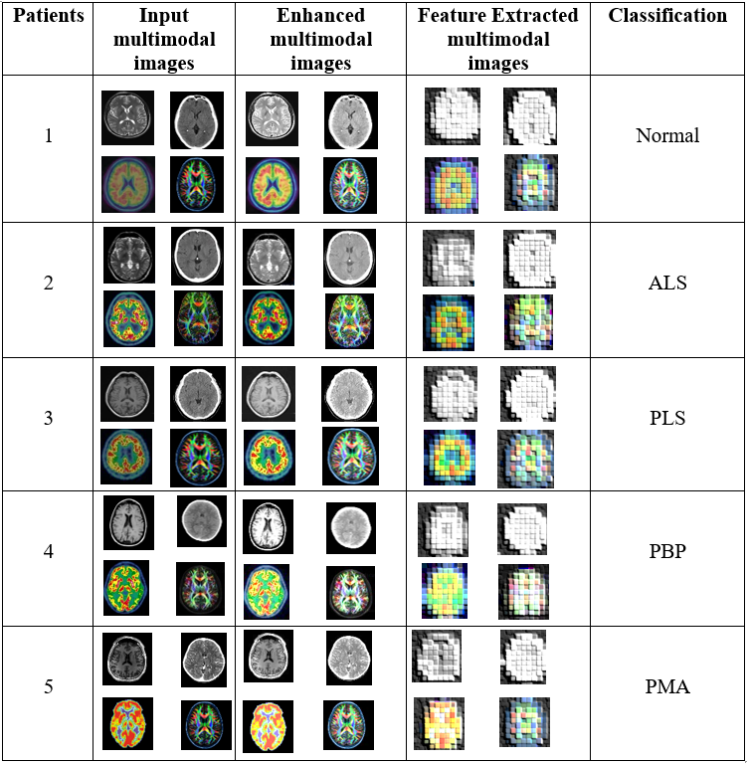
Experimental results of the proposed RES-MND method.

The experimental results of the proposed RES-MND method are illustrated in Figure 4. The patient identifiers are listed in column 1. The input multimodal images of the patients are shown in column 2. The enhanced multimodal images shown in column 3 were preprocessed to improve image quality. The feature-extracted multimodal images are presented in column 4. Finally, the classification results obtained using the proposed method are displayed in column 5.

### 3.1. Performance analysis

The experimental results demonstrate the performance characteristics of MND recognition, including precision, sensitivity, specificity, accuracy, recall, and F1-score.


(30) $$Accuracy=\frac{T_{R}P_{O}^{+}+T_{R}N_{E}^{+}}{T_{R}P_{O}^{+}+T_{R}N_{E}^{+}+f_{A}P_{O}^{+}+f_{A}N_{E}^{+}}\times 100$$

(31) $$Precision=\frac{T_{R}P_{O}^{+}}{T_{R}P_{O}^{+}+f_{A}P_{O}^{+}}$$

(32) $$Recall=\frac{T_{R}P_{O}^{+}}{T_{R}P_{O}^{+}+f_{A}P_{O}^{+}}$$

(33) $$F1-Score=\frac{2PrRe}{Pr+Re}$$

where *T_R_P_O_*^+^ and *T_R_N_E_*^+^ represent the true positive and true negative values of the input images, while *f_A_P_O_*^+^ and *f_A_N_E_*^+^ correspond to the false positive and false negative predictions, respectively.

The proposed RES-MND model achieved accuracy, recall, precision, and F1-score values of 99.35%, 93.03%, 87.61%, and 96.86%, respectively.

**Figure 5:**
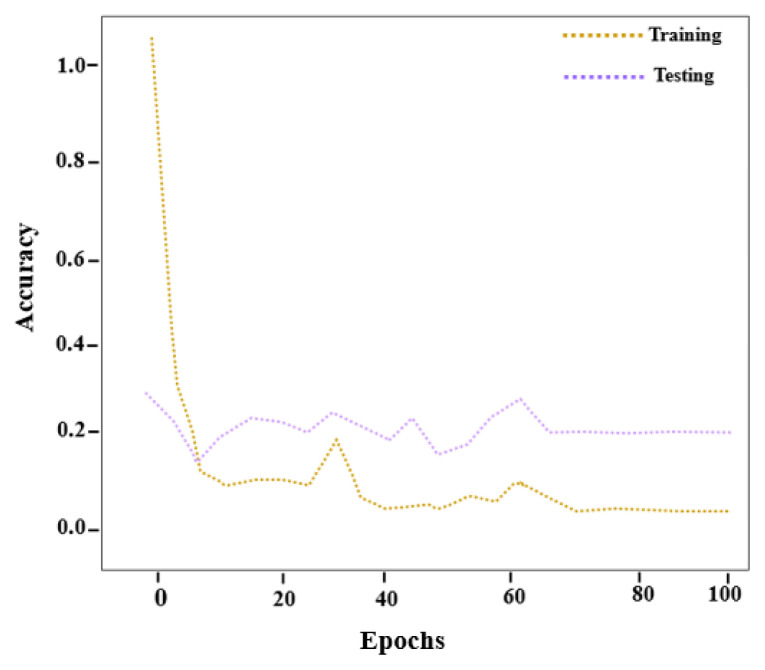
Accuracy curve of the proposed RES-MND method.

The accuracy curve shown in Figure 5 is based on 100 training epochs within a predefined accuracy range. The proposed disease detection model achieves higher accuracy as the number of training epochs increases.

**Figure 6:**
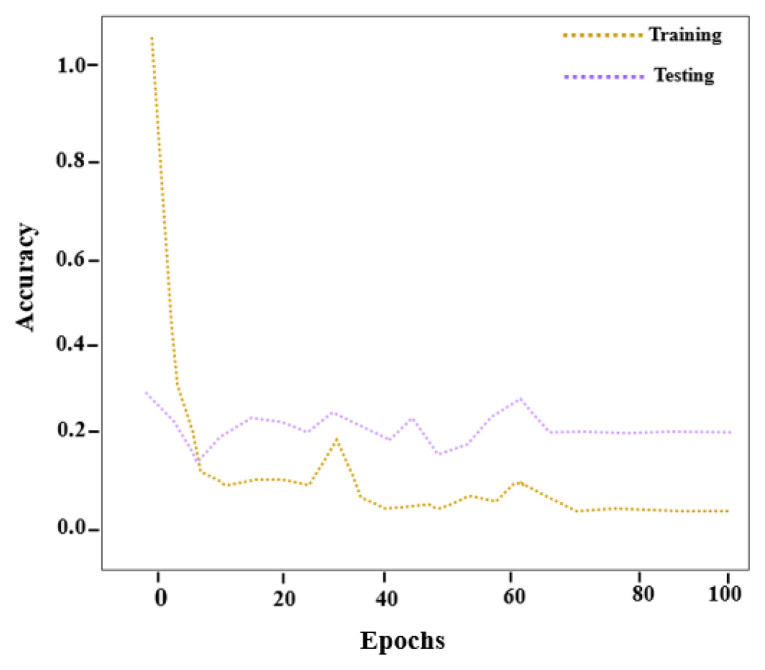
Loss curve of the proposed RES-MND method.

Figure 6 illustrates the reduction in loss as the proposed model progresses through successive training epochs. The figure presents the relationship between the number of epochs and the corresponding loss values. After 100 training epochs, the proposed model achieved a detection accuracy of 99.35% with a minimal error rate.

### 3.2. Comparative analysis

This section presents a comparative analysis between the proposed method and existing neural network models. The performance of the proposed approach was evaluated using standard metrics such as F1-score, accuracy, precision, and recall.

Table 1 presents a comparison between the proposed Res4Net-CBAM model and other conventional deep neural networks. The proposed Res4Net-CBAM achieves the highest accuracy rate compared with the other traditional networks. It improves the overall accuracy by 0.68%, 2.36%, 0.48%, and 0.89% compared to GoogleNet, ShuffleNet, AlexNet, and MobileNet respectively.

**Figure 7:**
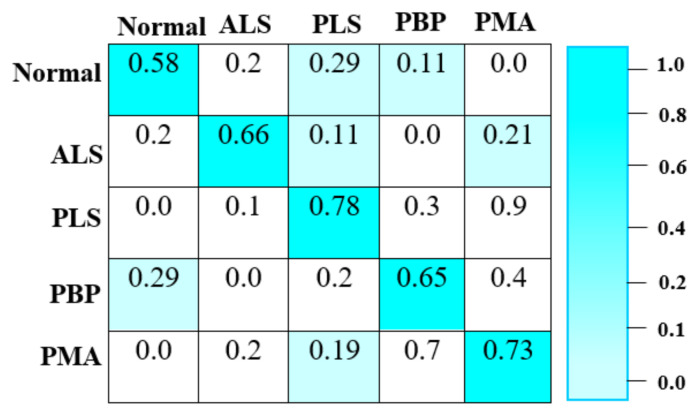
Confusion matrix of the proposed RES-MND model.

The results of the proposed model were obtained after an extensive trial period, and the five-class classification confusion matrix is presented in Figure 7. The proposed model accurately identifies all five MND categories—normal, ALS, PLS, PBP, and PMA—without any missed detections. Based on the confusion matrix, the proposed model demonstrates a lower misclassification rate and high classification accuracy in MND detection.

Table 2 compares several standard DL networks to identify the model with the highest classification accuracy. Compared to the proposed model, the traditional DL networks failed to produce improved outcomes. The proposed DBN achieved accuracy improvements of 0.68%, 0.41%, and 0.9% over spiking neural network (SNN), deep neural network (DNN), and CNN, respectively.

Table 3 presents an accuracy comparison between the proposed RES-MND method and the existing miRNA-, vGRF-, and SVM-RFE–based methods. The proposed RES-MND method improves accuracy by 1.08%, 2.18%, and 1.7% compared to the existing methods. The proposed model achieves an overall accuracy of 99.56%, which is higher than that of the other models. However, the existing networks were less reliable in terms of error rate compared to the proposed model.

## 4. Discussion

The proposed RES-MND model is designed to detect MND using multimodal medical images. The experimental results of the proposed method are illustrated in Figure 4. Table 1 presents a comparison between the proposed Res4Net-CBAM feature extraction framework and other conventional deep neural networks. Figure 7 displays the five-class classification confusion matrix generated after an extensive trial period. Table 2 compares the proposed DBN-based classification network with other traditional models. Table 3 presents an accuracy comparison between the proposed RES-MND method and the existing miRNA-, vGRF-, and SVM-RFE–based methods. The performance of the proposed RES-MND method was evaluated using standard metrics such as accuracy, precision, recall, and F1-score. According to the results, the proposed RES-MND method achieved the highest accuracy rate of 99.56%, outperforming all existing methods.

The input multimodal images, including MRI, CT, PET, and DTI scans, were preprocessed using the adaptive dynamic histogram equalization (ADHE) and the total variation bilateral filter (TVBF). The preprocessed multimodal images were then processed through the Res4Net-CBAM framework for feature extraction to enhance image recognition performance. A dove swarm optimization algorithm was employed to select the most relevant features from the multimodal images. Finally, the deep belief network (DBN) classified five MND categories—normal, ALS, PLS, PBP, and PMA. The proposed DBN achieved accuracy improvements of 0.68%, 0.41%, and 0.9% over SNN, DNN, and CNN, respectively. It also improved overall accuracy by 1.08%, 2.18%, and 1.7% compared to the existing methods, including miRNA-, vGRF-, and SVM-RFE–based approaches. In the future, the RES-MND model can be made more robust and generalizable by expanding the dataset and integrating additional advanced machine learning techniques.

## Figures and Tables

**Table 1:** Comparison between the proposed method and other neural network models.

Network	Accuracy	Precision	Recall	F1-Score
**GoogleNet**	98.97%	90.84%	76.46%	92.26%
**ShuffleNet**	97.29%	91.64%	81.15%	89.84%
**ResNet**	99.17%	85.88%	84.54%	92.62%
**MobileNet**	98.76%	89.28%	77.78%	94.90%
**Res4Net-CBAM (Proposed Method)**	99.65%	93.03%	87.61%	96.86%

**Table 2:** Comparison between existing deep learning methods and the proposed model.

Method	Accuracy	Precision	Recall	F1-Score
**SNN**	98.97%	87.64%	85.83%	90.42%
**DNN**	99.24%	90.58%	79.94%	88.52%
**CNN**	98.75%	88.19%	82.79%	87.59%
**DBN**	99.65%	93.03%	87.61%	96.86%

**Table 3:** Accuracy comparison between existing approaches and the proposed RES-MND method.

Author	Method	Accuracy
Banack et al. [19]	miRNA	98.57%
Setiawanand Lin[20]	vGRF	97.47%
Greco et al. [21]	SVM-RFE	97.95%
Proposed Method	RES-MND	99.65%
